# The Role of Hemosiderin Excision in Seizure Outcome in Cerebral Cavernous Malformation Surgery: A Systematic Review and Meta-Analysis

**DOI:** 10.1371/journal.pone.0136619

**Published:** 2015-08-25

**Authors:** Di Ruan, Xiao-Bo Yu, Sudeep Shrestha, Lin Wang, Gao Chen

**Affiliations:** Department of Neurosurgery, the Second Affiliated Hospital of Zhejiang University School of Medicine, Hangzhou, the People’s Republic of China; Sainte-Anne Hospital Center, FRANCE

## Abstract

**Background and Purpose:**

Whether the excision of hemosiderin surrounding cerebral cavernous malformations (CCMs) is necessary to achieve a seizure-free result has been the subject of debate. Here, we report a systematic review of related literature up to Jan 1, 2015 including 594 patients to assess the effect of hemosiderin excision on seizure outcome in patients with CCMs by meta-analysis.

**Methods:**

Ten studies comparing extended hemosiderin excision with only lesion resection were identified by searching the English-language literature. Meta-analyses, subgroup analyses and sensitivity analysis were conducted to determine the association between hemosiderin excision and seizure outcome after surgery.

**Results:**

Seizure outcome was significantly improved in the patients who underwent an extended excision of the surrounding hemosiderin (OR, 0.62; 95% CI: 0.42–0.91; *P* = 0.01). In subgroup analysis, studies from Asia (OR, 0.42; 95% CI: 0.25–0.71; *P* = 0.001), male-majority (female ratio < 50%) studies (OR, 0.56; 95% CI: 0.33–0.96; *P* = 0.04), low occurrence rate of multiple CCMs (OR, 0.37; 95% CI: 0.20–0.71; *P* = 0.003), cohort studies (OR, 0.44; 95% CI: 0.28–0.68; *P* = 0.78), longer duration of seizure symptoms (> 1 year) before surgery (OR, 0.43; 95% CI: 0.22–0.84; *P* = 0.01), lesion diameter > 2 cm (OR, 0.41; 95% CI: 0.19–0.87; *P* = 0.02) and short-term (< 3 years) follow-up (OR, 0.48; 95% CI: 0.29–0.80; *P* = 0.005) tended to correlate with a significantly favorable outcome.

**Conclusion:**

Patients who underwent extended surrounding hemosiderin excision could exhibit significantly improved seizure outcomes compared to patients without hemosiderin excision. However, further well-designed prospective multiple-center RCT studies are still needed.

## Introduction

Seizures are the most common frequent clinical symptom caused by cerebral cavernous malformations (CCMs), presenting in 23%-79% of CCM patients [1–3]. Among these patients, approximately 40% of individuals progress to medically refractory epilepsy, which can dramatically decrease quality of life due to various disabilities [4]. Although the remaining 60% of patients with CCM may benefit from antiepileptic drugs (AEDs), they usually suffer the unfavorable side effects of AEDs [5, 6]. Currently, it is widely accepted that the surgical resection of CCMs is the best treatment strategy for patients with medically refractory epilepsy [7–10]. CCMs are often surrounded by hemosiderin, which has been suggested to produce seizures [11–13]. However, whether the excision of hemosiderin surrounding CCMs is necessary to achieve a seizure-free result has been under debate [14].

Some articles analyzing various predictors of seizure freedom in the surgical treatment of CCMs have been published, but none have specifically focused on hemosiderin excision alone. Among these clinical reports, some supported the idea that extended resection of hemosiderin might improve short-term or long-term seizure outcomes [15–18], but others showed no significant differences between the excision of the hemosiderin along with the lesion and resection of the cavernoma only [19–21]. Given these contradictory reports and the theoretical potential for additional morbidity with extended cortical resection, it is important to systematically evaluate the role of hemosiderin in CCM surgery for clinical treatment [14].

Here, we report a systematic review of related literature up to Jan 1, 2015. Our purposes are as follows: (1) to assess the effect of hemosiderin excision on seizure outcome in patients with CCMs by meta-analysis; (2) to identify the factors influencing our result using subgroup analysis; and (3) to provide some evidence for clinical decision-making.

## Methods

This meta-analysis was performed in accordance with the PRISMA 2009 checklist. (data in S1 Checklist)

### 1. Search strategy and study identification

Appropriate studies relevant to the effect of hemosiderin excision on seizure outcome in patients with CCM surgery were identified by searching online databases: PubMed, Web of Science and EBSCO. The following key words were used and connected by Boolean logic operators: “cavernous hemangioma”, “cavernous angioma”, “angiocavernoma”, “hemangioma”, “cavernous”, “hemosiderin ring”, “hemosiderin”, “epilepsy”, “seizure”, “surgery”. Searches were restricted to the English-language literature but were not limited with regard to the publication year. Three authors (Di Ruan, Xiao-Bo Yu and Sudeep Shrestha) were independently responsible for checking and selecting articles, with disagreements settled by the senior authors (Lin Wang and Gao Chen). Reference lists in the identified publications were also examined to find additional studies.

### 2. Inclusion and exclusion criteria

**Fig 1:**
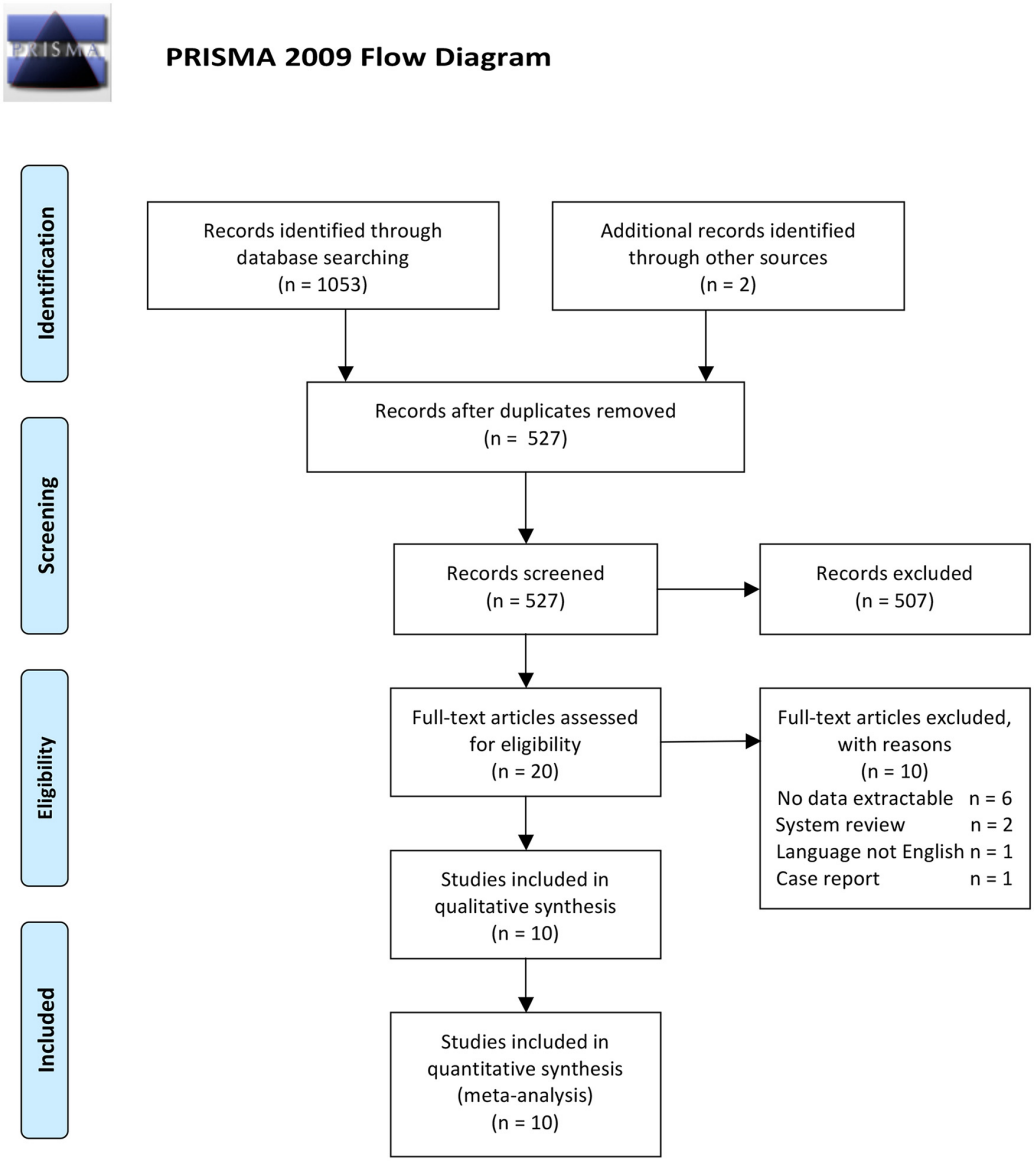
Flow chart of literature selection.

Fig 1 details the selection criteria we used. The inclusion criteria were as follows: (1) series of at least 10 patients undergoing CCM surgery; (2) cohort or case-control studies comparing the extended hemosiderin excision with lesion resection only; (3) CCM patients with epilepsy or seizure symptoms before surgery; (4) seizure outcomes measured or calculated according to Engel Class; (5) explicitly reported numbers of patients who underwent or did not undergo hemosiderin excision; (6) duration of follow-up of at least 12 months; and (7) study quality score >4 on the Newcastle-Ottawa Scale (NOS) [22]. The exclusion criteria were as follows: (1) study did not provide sufficient extractable data on the patient number and follow-up outcome; (2) study was a system review or case report; (3) study did not compare the seizure outcomes of the excision group and control group; (4) study was not written in English; and (5) study with only the abstract available, or unpublished study. The quality of case-control or cohort studies was assessed by the NOS.

### 3. Data extraction

Data were extracted independently by three authors (Di Ruan, Xiao-Bo Yu and Sudeep Shrestha) using a uniform standardized and digitized data extraction form in Excel 2010 and checked by these three authors until agreement was reached. The primary surgical outcome was the patient seizure status (seizure freedom (Engel ClassI) versus persistent seizures (Engel ClassII-IV)) after some period of follow-up. Although we are aware that the Engel classification has its disadvantages, we had to use it because almost all the related studies used this classification [23]. If a study did not report sufficient information for the calculation of the Engel Class, attempts were made to contact the authors of the articles, who were asked to provided either the Engel Class data or the raw data necessary for calculation. Data on other related factors data, such as age, sex, area, seizure duration before surgery, multiple CCM occurrence, lesion location, lesion size, follow-up time, study quality and study design type, were also extracted.

### 4. Statistical analysis

Meta-analyses and subgroup analysis were performed using Review Manager 5.3. Dichotomous variables were presented as odds ratios (OR; with hemosiderin excision (hemosiderin (-)) versus without hemosiderin excision (hemosiderin (+)). Heterogeneity was evaluated by the I^2^ value. A fixed effect model was used if the I^2^ value was less than 50%; otherwise, a random effect model was adopted. We set significance at *P* = 0.05. In addition to visual inspection of funnel plots using RevMan 5.3, the STATA 13.0 software was also used to perform the Begg’s test [24] and Egger’s test [25] methods to detect potential publication bias. Moreover, sensitivity analysis was performed using STATA 13.0.

## Results

### 1. Literature search findings and included publication characteristics

The search process and results are illustrated in the flowchart (Fig 1). We found a total of 1055 papers, with 527 remaining after duplicates were removed. Of this group, 507 were excluded based on reviewing the title and abstract. For the remaining 20 articles, the full text was accessed, and 10 articles met the inclusion criteria. Ten of the 20 papers were excluded because they lacked extractable data (n = 6), were system reviews (n = 2), were not written in English (n = 1), or were a case report (n = 1). Finally, 10 articles were included in the meta-analysis. [15, 17–21, 26–29]

### 2. Baseline characteristics of included studies

Ten articles containing 13 studies were obtained, including 4 case-control and 9 cohort studies. All studies had at least moderate-level quality using the Newcastle-Ottawa Scale evaluation system. The baseline characteristics of the studies are summarized in Table 1. The 13 included studies reported the outcomes of 594 patients with more than 632 CCMs ranging from 0.4 cm to 5.3 cm in diameter. In total, 234 of 316 patients in the hemosiderin (-) group and 189 of 278 in the hemosiderin (+) group were Engel ClassI.

**Table 1:** Baseline characteristics of included studies.

Study	Country	Patient number	Female n (%)	Mean age (yrs)	Mean size of the lesion (cm)	Temporal lobe n (%)	Multiple CCMs n (%)	Mean duration before surgery (mo)	Mean follow-up duration (mo)	Chg Engel Class 1	Chg Engel Class 2–4	Khg Engel Class 1	Khg Engel Class 2–4	Study design	Quality score
Huang C, 2013	China	27	8 (29.6)	NA	NA	16 (59.3)	14 (51.9)	NA	24.8	9	3	12	3	cc	7
Baumann CR, 2006	SI	31	15 (48.4)	36.3	1.8	20 (64.5)	1 (3.2)	144	12	11	3	11	6	cohort	6
Baumann CR, 2006	SI	29	NA	NA	NA	NA	NA	NA	24	8	5	7	9	cohort	6
Baumann CR, 2006	SI	27	NA	NA	NA	NA	NA	NA	36	11	3	7	6	cohort	6
von der Brelie C, 2013	Germany	11	4 (36.4)	15.3	NA	5 (45.5)	3 (27.2)	57.8	132.5	6	3	1	1	cohort	5
Kwon CS, 2013	USA	56	29 (51.8)	37.5	1.6	27 (48.2)	9 (16.1)	12*	87.9	23	8	23	2	cc	9
Zevgaridis D, 1996	Switzerland	66	NA	NA	NA	NA	NA	NA	39	24	4	33	5	cohort	6
Yeon JY, 2009	Korea	54	NA	NA	NA	NA	NA	NA	> 12	26	5	19	4	cohort	6
Cappabianca P, 1997	Italy	35	21 (60.0)	28.8	NA	10 (28.6)	NA	NA	> 24	4	0	25	6	cc	7
Hammen T, 2007	Germany	30	13 (42.3)	39.4	NA	21 (70.0)	4 (13.3)	130.8	> 48	4	7	12	7	cc	6
Wang X, 2013	China	132	64 (48.5)	39.3	2.9	51 (38.6)	7 (5.3)	NA	12	64	22	25	21	cohort	7
Wang X, 2013	China	60	NA	NA	NA	NA	NA	NA	60	25	17	5	13	cohort	7
Jin Y, 2014	China	36	15 (41.7)	37.8	1.9	26 (72.2)	0 (0.0)	5.95	18*	19	2	9	6	cohort	9

CCMs, cerebral cavernous malformations; mo, month; yrs, years; NA, not available; SI, Switzerland and Italy; cc, case-control; Chg, Cut hemosiderin group; Khg, Keep hemosiderin group.

*The data was presented in the median form.

### 3. Meta-analysis and sensitivity analysis

**Fig 2:**
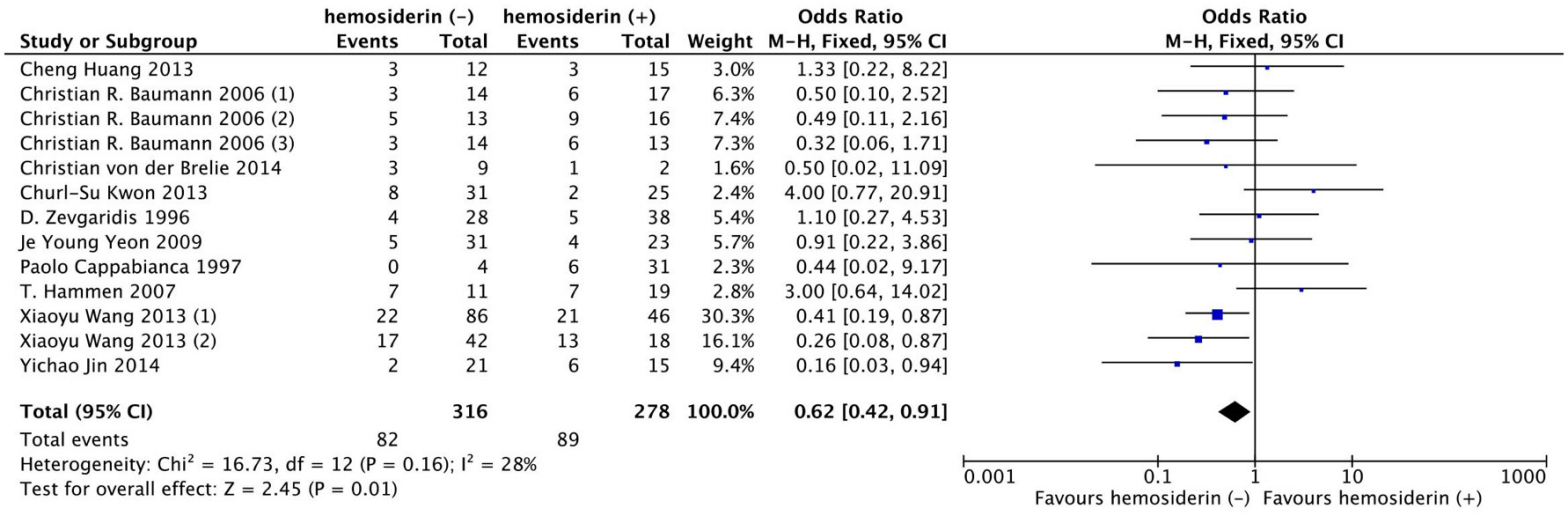
Forest plot of seizure outcomes comparing hemosiderin (-) group and hemosiderin (+) group. hemosiderin (-), with hemosiderin excision; hemosiderin (+), without hemosiderin excision; CI, confidence interval.

**Fig 3:**
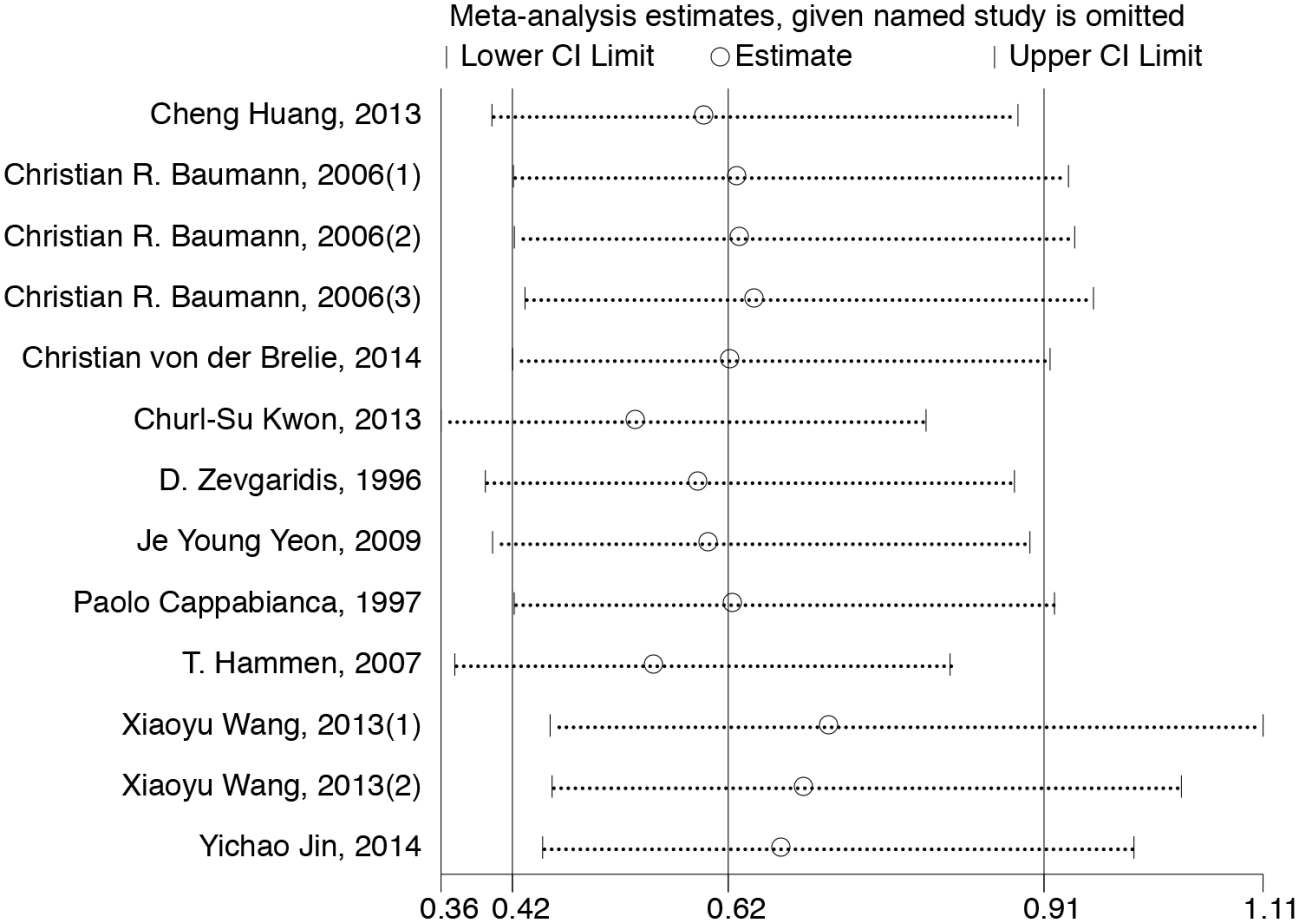
Sensitivity analysis of 13 included studies. CI, confidence interval.

Thirteen studies with a total of 594 patients reported the seizure outcomes after CCM surgeries (234 of 316 obtained Engel ClassI in the hemosiderin (-) group versus 189 of 278 in the hemosiderin (+) group). The seizure outcome was statistically significantly improved in the patients who underwent extended excision of the surrounding hemosiderin (OR, 0.62; 95% CI: 0.42–0.91; *P* = 0.01; I^2^ = 28%; Fig 2). Sensitivity analysis showed that the results of the association between hemosiderin excision and seizure outcome were robust, which demonstrated that no significant heterogeneity existed across the studies. (Fig 3)

### 4. Subgroup analysis

**Fig 4:**
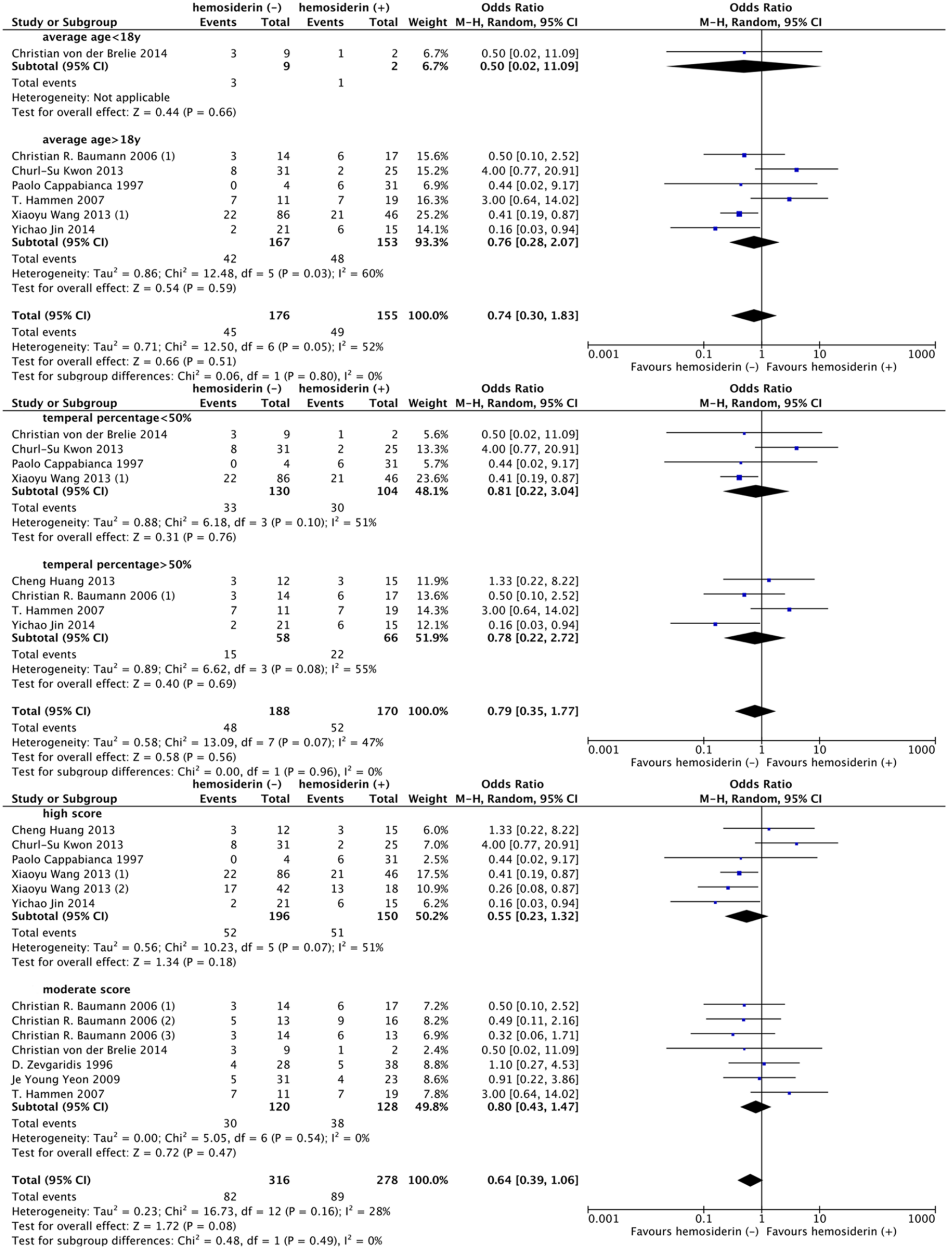
Forest plot comparing seizure outcomes between hemosiderin (+) and hemosiderin (-) groups in age, lesion location and study quality subgroup analyses. hemosiderin (-), with hemosiderin excision; hemosiderin (+), without hemosiderin excision; CI, confidence interval.

Although no heterogeneity was found between studies, we still performed subgroup analysis based on our clinical and statistical practice. Thus, age, sex, area, seizure duration before surgery, multiple CCM occurrence, lesion location, lesion size, follow-up time, study quality and study design type were considered as confounding factors in our studies. Among all these factors, there was no significant difference in age, lesion location and study quality level between the two groups according to our classification criteria. (Fig 4)

**Fig 5:**
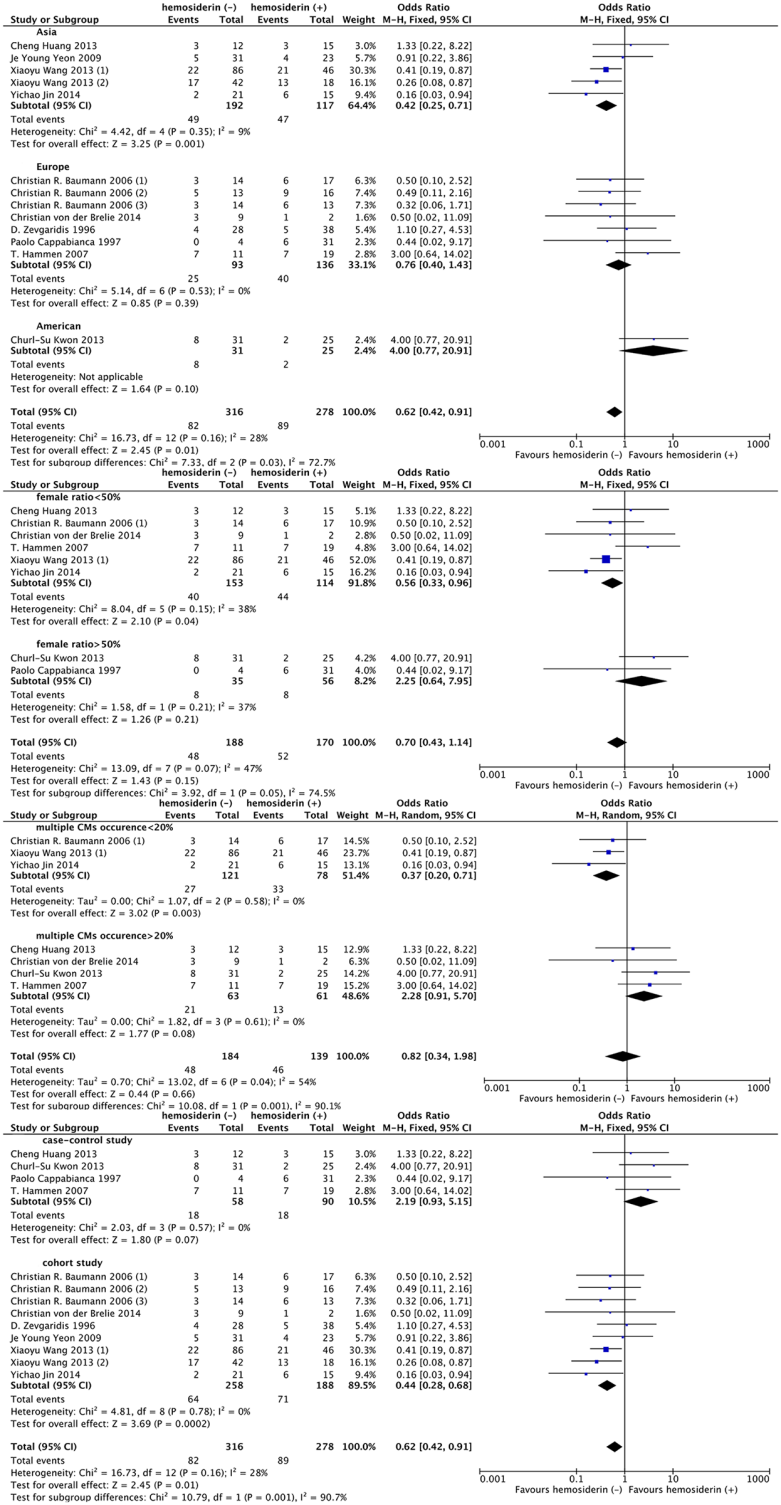
Forest plot comparing seizure outcomes between hemosiderin (+) and hemosiderin (-) groups in country, female ratio, occurrence of multiple CCMs and study design subgroup analyses. hemosiderin (-), with hemosiderin excision; hemosiderin (+), without hemosiderin excision; CI, confidence interval.

The studies from Asian countries reported a significantly higher rate of favorable outcomes in the hemosiderin (-) group (OR, 0.42; 95% CI: 0.25–0.71; *P* = 0.001; I^2^ = 9%). The male-majority studies tended to report a favorable outcome in the hemosiderin (-) group (OR, 0.56; 95% CI: 0.33–0.96; *P* = 0.04; I^2^ = 38%). The advantage of hemosiderin excision was obvious in the group with a low occurrence rate of multiple CCMs (OR, 0.37; 95% CI: 0.20–0.71; *P* = 0.003; I^2^ = 0%) but not obvious in the higher occurrence rate group (OR, 2.28; 95% CI: 0.91–5.70; *P* = 0.08; I^2^ = 0%). For different study types, cohort studies were inclined to report favorable outcomes in the hemosiderin (-) group (OR, 0.44; 95% CI: 0.28–0.68; *P* = 0.78; I^2^ = 0%). (Fig 5)

**Fig 6:**
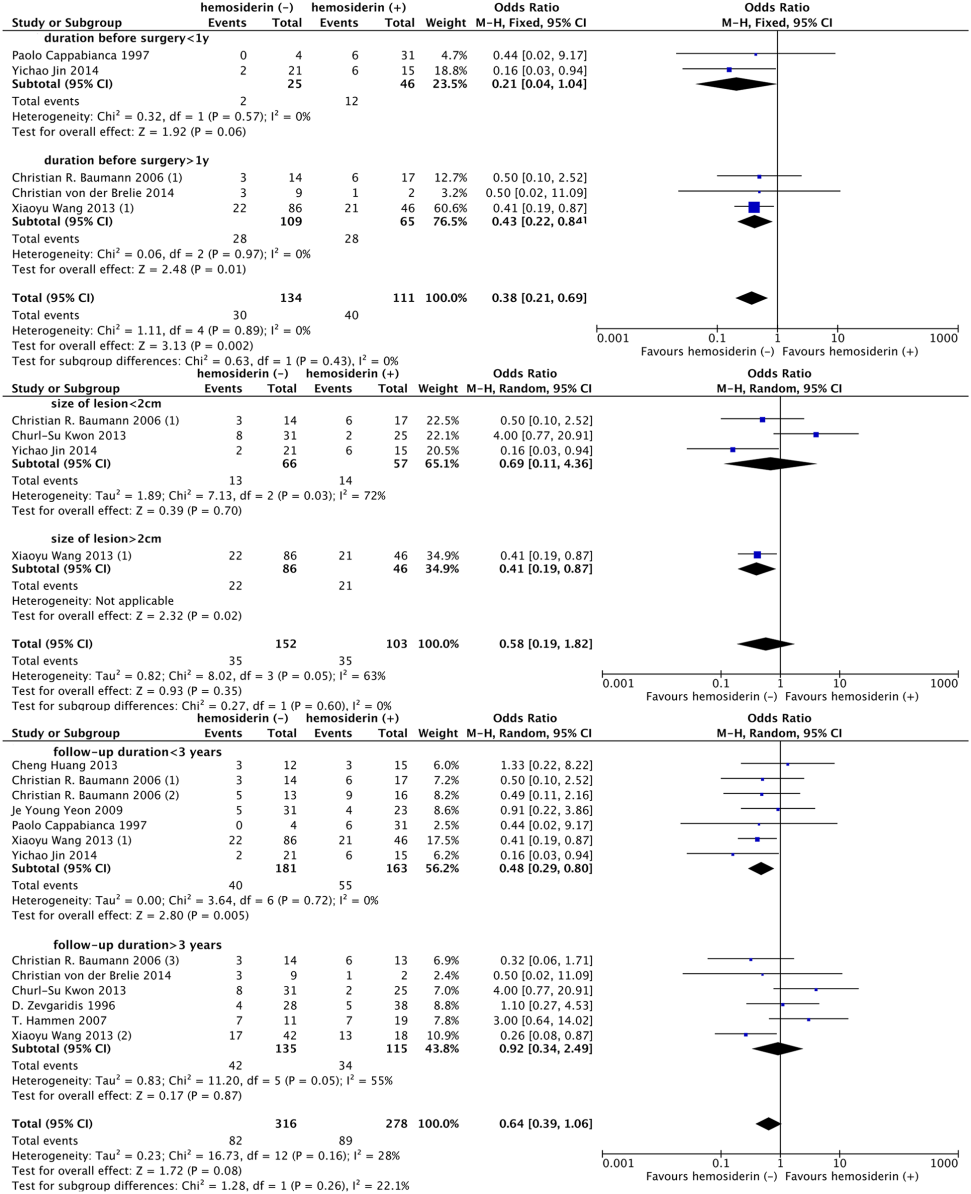
Forest plot comparing seizure outcomes between hemosiderin (+) and hemosiderin (-) groups in duration before surgery, lesion size and follow-up duration subgroup analyses. hemosiderin (-), with hemosiderin excision; hemosiderin (+), without hemosiderin excision; CI, confidence interval.

Regarding the seizure duration before surgery, lesion size and follow-up time, patients with seizure duration before surgery > 1 year (OR, 0.43; 95% CI: 0.22–0.84; *P* = 0.01; I^2^ = 0%), lesion diameter > 2 cm (OR, 0.41; 95% CI: 0.19–0.87; *P* = 0.02) and short-term (< 3 years) follow-up (OR, 0.48; 95% CI: 0.29–0.80; *P* = 0.005; I^2^ = 0%) appeared to have a more favorable outcome tendency in the hemosiderin (-) group. However, the benefit of hemosiderin excision was not significantly different from that gained by patients with seizure duration before surgery < 1 year (*P* = 0.43; I^2^ = 0%), lesion diameter < 2 cm (*P* = 0.60; I^2^ = 0%) and long-term (> 3 years) follow-up (*P* = 0.26; I^2^ = 22.1%), respectively. (Fig 6)

### 5. Publication bias

**Fig 7:**
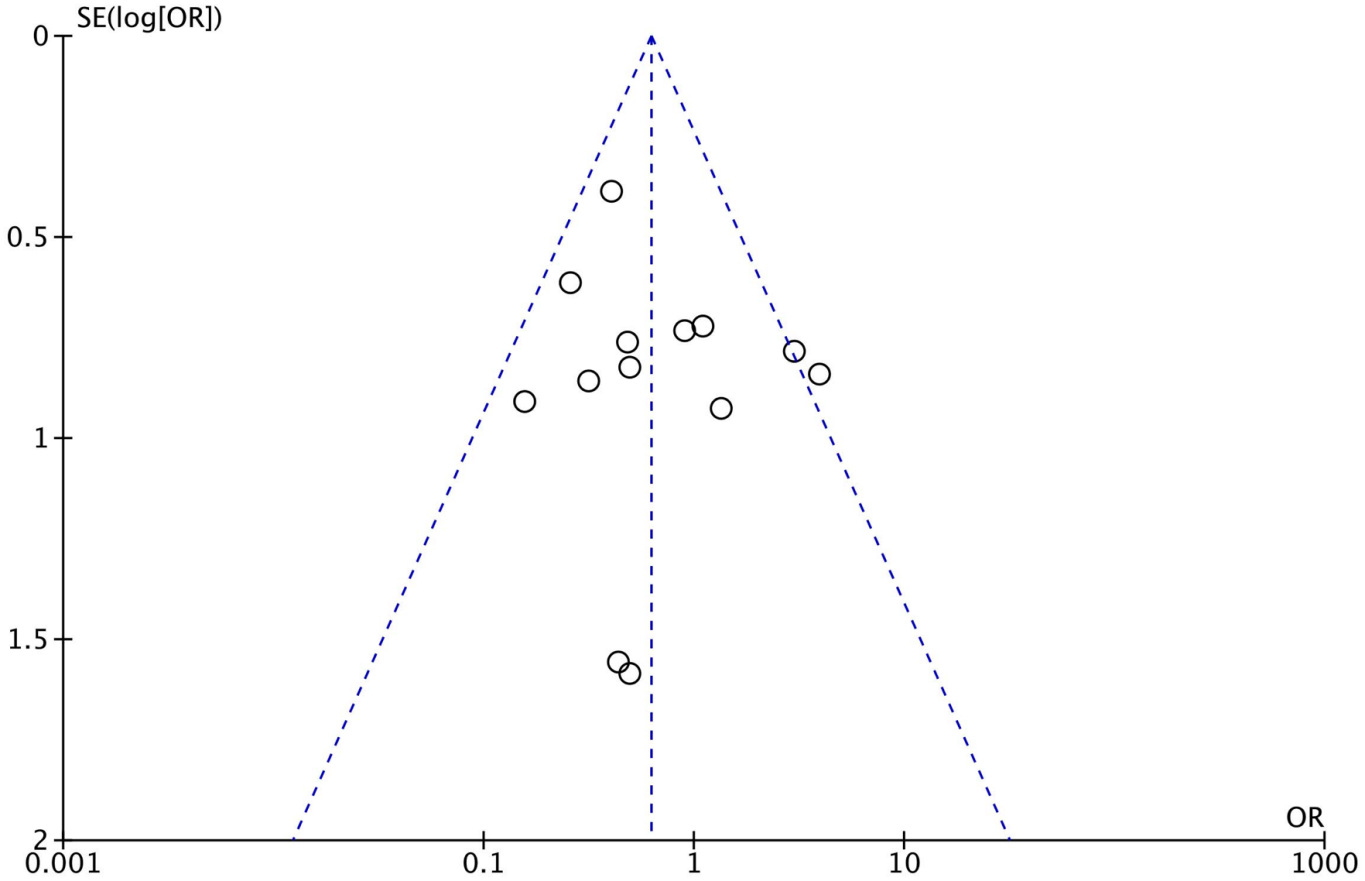
Funnel plot of publication bias. SE, standard error; OR, odds ratio.

No publication bias was found in a funnel plot, with plots visually symmetrically distributed along the vertical axis (Fig 7). Begg’s test and Egger’s test also showed no significant publication bias (Begg’s test, z = 0.48, *P* = 0.631; Egger’s test, t = 0.95, *P* = 0.367).

## Discussion

Here, we present the first meta-analysis of hemosiderin excision on seizure outcome in cerebral cavernous malformations surgery. The results of this paper show that seizure outcome was statistically significantly improved in patients who underwent an extended excision of the surrounding hemosiderin. However, there were many confounding factors that could influence the results. Thus, subgroup analyses were conducted to analyze the outcome more thoroughly. Among all the factors analyzed, studies from Asia, male majority (female ratio < 50%), low multiple CCM occurrence (< 20%) and cohort studies tended to correlate with a more favorable outcome in the hemosiderin excision group.

Regional differences existed among studies. However, we could easily observe that there was only one American article [27] concentrating on this topic. Thus, more non-Asian studies should be introduced to draw a more accurate conclusion. Regarding gender, the male-majority studies tended to report a favorable outcome in the hemosiderin (-) group, while the female-majority studies did not report an obvious result. However, this finding must be interpreted carefully because the sample size of the female group is smaller than that of the male group. For the lesion itself, low occurrence of multiple CCMs was prone to exhibit significant improvement, which might indicate that hemosiderin excision could be facilitated in patients with single CCMs. When focusing on study design type, we observed that cohort studies tended to report a more favorable outcome in the hemosiderin excision group, which increased the credibility of our meta-analysis result, as the cohort studies could justify or control for more confounders than case-control studies.

Patients with seizure duration before surgery > 1 year, lesion diameter > 2 cm and short-term (< 3 years) follow-up appeared to have a more favorable outcome tendency in the hemosiderin (-) group. However, we could not easily draw a conclusion that these kinds of patients could obtain a better prognosis after hemosiderin excision, because the benefit of hemosiderin excision was not significantly different from that gained by patients with seizure duration before surgery < 1 year, lesion diameter < 2 cm and long-term (> 3 years) follow-up, respectively. Thus, larger prospective trials are necessary to determine whether seizure duration before surgery, lesion size and follow-up time are significant confounding factors.

One previous system review [4] focused on the predictors of seizure outcome in the surgical treatment of cavernous malformations where hemosiderin excision was selected as one of the predictors. This review included a total of 763 CCM patients from 1985 to 2011 and stated that extended resection of the hemosiderin ring was not significantly predictive. The difference might be caused by the following reasons. First, the statistical method was different: Englot DJ et al. [4] used regression, while we used meta-analysis, allowing us to concentrate more on specific factors. Second, in our study, half of the articles were published after 2013, and the article sample was more precise because we only chose papers related to hemosiderin excision. Third, the significant improvement in seizure outcome after hemosiderin excision might result from recent advances in neurosurgery, allowing clinical excision to be performed more accurately and less invasively.

Up to now, the detail steps of epilepsy development in CCM has not been comprehended completely. However, chronic silent microhemorrhages were thought to be the main culprit [1, 28, 30–33]. When deposited in adjacent brain parenchyma, hemosiderin, a degradation product of blood, could produce free radicals and lipid peroxides to cause excitotoxicity of adjacent neurons and proliferation of the glial tissue by interrupting receptor activity, calcium hemostasis and neurotransmitter (glutamate, aspartate and phosphorylethanolamine) levels [11, 13, 30, 34, 35]. This mainstream hypothesis of the epileptogenicity of hemosiderin provided strong support to our results.

The shortcomings of this meta-analysis were as follows. First, the assessment methods of the complete removal of the hemosiderin ring were different between studies. Most of the included studies assessed the complete removal of the hemosiderin ring by post-operative MRI, while four studies [16, 18, 19, 26] used surgical records to determine whether hemosiderin deposits had been resected as postoperative MRI findings were not available. It was generally known that the surgical description of hemosiderin removal might differ from postoperative MRI controls, so we’d better use post-operative MRI, which could reflect hemosiderin ring excision more objectively, as the assessment criteria. Second, hemosiderin excision was only one of the factors related to seizure outcome in most studies, and only three articles [15, 17, 29] directly compare a hemosiderin excision group and a control group. Therefore, the patient baseline characteristics for the hemosiderin (+) group and hemosiderin (-) group were usually unavailable or incomplete, leading to the loss of some evidence in the subgroup analysis. Third, we only included studies using Engel I rather than Engel Ia (e.g., Stavrou I [16]) as the outcome index, which might omit some meaningful evidence. However, the Engel classification is a common clinical method in evaluating seizures. Fourth, as we searched only studies in English, some potential reports not written in English (e.g., Stefan H [36]) might have been missed. Fifth, many factors could influence the seizure state of CCM patients after operation, but we only focused on whether the excision of hemosiderin is necessary to achieve seizure freedom, which is currently uncertain. Sixth, all the data we extracted were from observational studies, as no randomized control trials (RCTs) were yet available; therefore, more convincingly designed studies are urgently needed. In summary, for a better answer to this question, a well-designed prospective multiple-center randomized control trial should be performed.

## Conclusions

This meta-analysis compared hemosiderin excision and hemosiderin reservation groups in CCM surgery and demonstrated that seizure outcome could be significantly improved in patients who undergo extended excision of the surrounding hemosiderin.

## Supporting Information

S1 Checklist PRISMA 2009 checklist. (DOC)

## References

[1] Chang EF, Gabriel RA, Potts MB, Garcia PA, Barbaro NM, Lawton MT. Seizure characteristics and control after microsurgical resection of supratentorial cerebral cavernous malformations. Neurosurgery. 2009 7;65(1):31–7; discussion 7–8. 10.1227/01.NEU.0000346648.03272.07 19574823

[2] Moran NF, Fish DR, Kitchen N, Shorvon S, Kendall BE, Stevens JM. Supratentorial cavernous haemangiomas and epilepsy: a review of the literature and case series. J Neurol Neurosurg Psychiatry. 1999 5;66(5):561–8. 10209164 10.1136/jnnp.66.5.561 PMC1736368

[3] Kondziolka D, Lunsford LD, Kestle JRW. The natural history of cerebral cavernous malformations. Journal of Neurosurgery. 1995;83(5):822–4. 10.3171/jns.1995.83.5.0820 7472549

[4] Englot DJ, Han SJ, Lawton MT, Chang EF. Predictors of seizure freedom in the surgical treatment of supratentorial cavernous malformations. J Neurosurg. 2011 12;115(6):1169–74. 10.3171/2011.7.JNS11536 21819194

[5] Cascino GD. When drugs and surgery don't work. Epilepsia. 2008;49:79–84. 10.1111/j.1528-1167.2008.01930.x 19087121

[6] Cramer JA, Mintzer S, Wheless J, Mattson RH. Adverse effects of antiepileptic drugs: a brief overview of important issues. Expert Rev Neurother. 2010 6;10(6):885–91. 10.1586/ern.10.71 20518605

[7] Mehdorn HM, Barth H, Buhl R, Nabavi A, Weinert D. Intracranial cavernomas: Indications for and results of surgery. Neurol Med-Chir. 1998 10;38:245–9. 10.2176/nmc.38.suppl_245 10235013

[8] Noto S, Fujii M, Akimura T, Imoto H, Nomura S, Kajiwara K, et al Management of patients with cavernous angiomas presenting epileptic seizures. Surg Neurol. 2005 12;64(6):495–9. 16293460 10.1016/j.surneu.2005.03.045

[9] Van Gompel JJ, Marsh WR, Meyer FB, Worrell GA. Patient-assessed satisfaction and outcome after microsurgical resection of cavernomas causing epilepsy. Neurosurg Focus. 2010 9;29(3). 10.3171/2010.6.FOCUS10127 PMC3920597 20809757

[10] Baumann CR, Acciarri N, Bertalanffy H, Devinsky O, Elger CE, Lo Russo G, et al Seizure outcome after resection of supratentorial cavernous malformations: a study of 168 patients. Epilepsia. 2007 3;48(3):559–63. 17346251 10.1111/j.1528-1167.2006.00941.x

[11] vonEssen C, Rydenhag B, Nystrom B, Mozzi R, vanGelder N, Hamberger A. High levels of glycine and serine as a cause of the seizure symptoms of cavernous angiomas? Journal of Neurochemistry. 1996 7;67(1):260–4. 8667000 10.1046/j.1471-4159.1996.67010260.x

[12] Washington CW, McCoy KE, Zipfel GJ. Update on the natural history of cavernous malformations and factors predicting aggressive clinical presentation. Neurosurg Focus. 2010 9;29(3). 10.3171/2010.5.FOCUS10149 20809765

[13] Williamson A, Patrylo PR, Lee S, Spencer DD. Physiology of human cortical neurons adjacent to cavernous malformations and tumors. Epilepsia. 2003;44(11):1413–9. 14636349 10.1046/j.1528-1157.2003.23603.x

[14] Englot DJ, Chang EF. Rates and predictors of seizure freedom in resective epilepsy surgery: an update. Neurosurg Rev. 2014 7;37(3):389–404. 10.1007/s10143-014-0527-9 24497269 PMC5257205

[15] Wang X, Tao Z, You C, Li Q, Liu Y. Extended resection of hemosiderin fringe is better for seizure outcome: a study in patients with cavernous malformation associated with refractory epilepsy. Neurol India. 2013 May-Jun;61(3):288–92. 10.4103/0028-3886.115070 23860150

[16] Stavrou I, Baumgartner C, Frischer JM, Trattnig S, Knosp E. Long-term seizure control after resection of supratentorial cavernomas: a retrospective single-center study in 53 patients. Neurosurgery. 2008 11;63(5):888–96. 10.1227/01.NEU.0000327881.72964.6E 19005379

[17] Baumann CR, Schuknecht B, Lo Russo G, Cossu M, Citterio A, Andermann F, et al Seizure outcome after resection of cavernous malformations is better when surrounding hemosiderin-stained brain also is removed. Epilepsia. 2006 3;47(3):563–6. 16529622 10.1111/j.1528-1167.2006.00468.x

[18] von der Brelie C, Malter MP, Niehusmann P, Elger CE, von Lehe M, Schramm J. Surgical management and long-term seizure outcome after epilepsy surgery for different types of epilepsy associated with cerebral cavernous malformations. Epilepsia. 2013 9;54(9):1699–706. 10.1111/epi.12327 23944932

[19] Zevgaridis D, van Velthoven V, Ebeling U, Reulen HJ. Seizure control following surgery in supratentorial cavernous malformations: a retrospective study in 77 patients. Acta Neurochir (Wien). 1996;138(6):672–7. 8836281 10.1007/BF01411470

[20] Cappabianca P, Alfieri A, Maiuri F, Mariniello G, Cirillo S, de Divitiis E. Supratentorial cavernous malformations and epilepsy: seizure outcome after lesionectomy on a series of 35 patients. Clin Neurol Neurosurg. 1997 8;99(3):179–83. 9350398 10.1016/s0303-8467(97)00023-1

[21] Yeon JY, Kim JS, Choi SJ, Seo DW, Hong SB, Hong SC. Supratentorial cavernous angiomas presenting with seizures: surgical outcomes in 60 consecutive patients. Seizure. 2009 1;18(1):14–20. 10.1016/j.seizure.2008.05.010 18656386

[22] Wells GA, Shea B, O’Connell D, Peterson J, Welch V, Losos M, et al. The Newcastle-Ottawa Scale (NOS) for assessing the quality if nonrandomized studies in meta-analyses. Available from: http://www.ohri.ca/programs/clinical_epidemiology/oxford.htm [cited 2009 Oct 19].

[23] Wieser HG, Blume WT, Fish G, Goldensohn E, Hufnagel A, King D, et al Proposal for a new classification of outcome with respect to epileptic seizures following epilepsy surgery. Epilepsia. 2001 2;42(2):282–6. 11240604

[24] Begg CB, Mazumdar M. Operating characteristics of a rank correlation test for publication bias. Biometrics. 1994 12;50(4):1088–101. 7786990

[25] Egger M, Davey Smith G, Schneider M, Minder C. Bias in meta-analysis detected by a simple, graphical test. BMJ. 1997 9 13;315(7109):629–34. 9310563 10.1136/bmj.315.7109.629 PMC2127453

[26] Huang C, Chen MW, Si Y, Li JM, Zhou D. Factors associated with epileptic seizure of cavernous malformations in the central nervous system in West China. Pak J Med Sci. 2013 9;29(5):1116–21. 24353703 10.12669/pjms.295.3681 PMC3858921

[27] Kwon CS, Sheth SA, Walcott BP, Neal J, Eskandar EN, Ogilvy CS. Long-term seizure outcomes following resection of supratentorial cavernous malformations. Clin Neurol Neurosurg. 2013 11;115(11):2377–81. 10.1016/j.clineuro.2013.08.024 24075713

[28] Hammen T, Romstock J, Dorfler A, Kerling F, Buchfelder M, Stefan H. Prediction of postoperative outcome with special respect to removal of hemosiderin fringe: a study in patients with cavernous haemangiomas associated with symptomatic epilepsy. Seizure. 2007 4;16(3):248–53. 17276092 10.1016/j.seizure.2007.01.001

[29] Jin Y, Zhao C, Zhang S, Zhang X, Qiu Y, Jiang J. Seizure outcome after surgical resection of supratentorial cavernous malformations plus hemosiderin rim in patients with short duration of epilepsy. Clin Neurol Neurosurg. 2014 4;119:59–63. 10.1016/j.clineuro.2014.01.013 24635927

[30] Awad IA, Robinson JR Jr, Mohanty S, Estes ML. Mixed vascular malformations of the brain: clinical and pathogenetic considerations. Neurosurgery. 1993 8;33(2):179–88; discussion 188. 8367039 10.1227/00006123-199308000-00001

[31] Kraemer DL, Awad IA. Vascular malformations and epilepsy: clinical considerations and basic mechanisms. Epilepsia. 1994;35 Suppl 6:S30–43. 8206013 10.1111/j.1528-1157.1994.tb05987.x

[32] Bertalanffy H, Benes L, Miyazawa T, Alberti O, Siegel AM, Sure U. Cerebral cavernomas in the adult. Review of the literature and analysis of 72 surgically treated patients. Neurosurg Rev. Neurosurg Rev. 2002 3;25(1–2):1–53; discussion 54–5. 11954761 10.1007/s101430100179

[33] Kim W, Stramotas S, Choy W, Dye J, Nagasawa D, Yang I. Prognostic factors for post-operative seizure outcomes after cavernousmalformation treatment. J Clin Neurosci. 2011 7;18(7):877–80. 10.1016/j.jocn.2010.12.008 21561775

[34] Volterra A, Trotti D, Tromba C, Floridi S, Racagni G. Glutamate uptake inhibition by oxygen free radicals in rat cortical astrocytes. J Neurosci. 1994 5;14(5 Pt 1):2924–32. 7910203 10.1523/JNEUROSCI.14-05-02924.1994 PMC6577465

[35] Tymianski M, Tator CH. Normal and abnormal calcium homeostasis in neurons: a basis for the pathophysiology of traumatic and ischemic central nervous system injury. Neurosurgery. 1996 6;38(6):1176–95. 8727150 10.1097/00006123-199606000-00028

[36] Stefan H, Walter J, Kerling F, Blumcke I, Buchfelder M. Supratentorial cavernoma and epileptic seizures. Are there predictors for postoperative seizure control? Nervenarzt. 2004 8;75(8):755–62. 15221063 10.1007/s00115-004-1697-4

